# Advantages and Disadvantages of Reconstructive and Preservation Rhinoplasty: Surgical Techniques, Outcomes, and Future Directions

**DOI:** 10.7759/cureus.69002

**Published:** 2024-09-09

**Authors:** Christopher R Meretsky, Andreas Polychronis, David Clark, Dimitria Liovas, Anthony T Schiuma

**Affiliations:** 1 Surgery, St. George's University School of Medicine, Great River, USA; 2 General Surgery, St. George's University School of Medicine, Great River, USA; 3 Emergency Medicine, St. George's University School of Medicine, Great River, USA; 4 Medicine, St. George's University School of Medicine, Great River, USA; 5 Orthopedic Surgery, Holy Cross Hospital, Fort Lauderdale, USA

**Keywords:** 3d imaging, artificial intelligence, cartilage grafting, patient outcomes, preservation rhinoplasty, reconstructive rhinoplasty, surgical techniques, tissue engineering

## Abstract

Reconstructive rhinoplasty, a specialized surgical procedure, aims to restore both the form and function of the nose, particularly after trauma, congenital defects, or prior surgeries. This review evaluates the advantages and disadvantages of various surgical techniques used in reconstructive and preservation rhinoplasty. The study focuses on the outcomes of commonly employed methods such as cartilage grafting, flap techniques, and alloplastic materials, assessing both functional and aesthetic results. Recent advancements, including 3D imaging, tissue engineering, and artificial intelligence, are discussed as potential future directions that could enhance surgical precision, safety, and patient care. The review systematically examines clinical studies from the past decade, highlighting the evolving landscape of rhinoplasty and its impact on patient outcomes.

## Introduction and background

Structural (reconstructive) rhinoplasty involves surgical techniques that utilize either external (open) or internal (closed) incisions. This procedure includes the resection of bones, cartilage, and skin, followed by the reconstruction and reshaping of the nasal structure. It often involves the use of additional bone, cartilage, or skin grafts to achieve the desired structural and aesthetic outcomes [1]. Reconstructive rhinoplasty is a specialized surgical procedure designed to restore the form and function of the nose after trauma, congenital defects, or previous surgeries. This surgery is crucial not only for aesthetic reasons but also for restoring nasal function, particularly in cases where breathing is impaired [2]. The field has seen significant advancements in surgical techniques, including cartilage grafts, flap techniques, and alloplastic materials, which have greatly improved both functional and aesthetic outcomes [3]. In contrast, preservation rhinoplasty involves conservative techniques primarily aimed at enhancing the aesthetic appearance of the nose. However, it can also improve airway function in certain cases, such as addressing septal deviation or nasal valving issues, where the sidewalls of the nose collapse during inhalation [4].

Surgeons must consider the complexity of the nasal structure, which includes bone, cartilage, and soft tissue, each requiring precise manipulation [5]. The choice of technique often depends on the specific defect being addressed, patient anatomy, and the surgeon's expertise [6]. Outcomes of reconstructive rhinoplasty are measured not only by the appearance of the nose but also by the patient's ability to breathe effectively and the overall facial symmetry. Recent studies have shown promising results with the use of 3D imaging and printing technologies, which allow for better preoperative planning and customization of implants [7]. Additionally, ongoing research into the use of stem cells and tissue engineering aims to improve the regeneration of nasal tissues, potentially leading to more effective and less invasive procedures in the future [8]. Preservation rhinoplasty describes surgical techniques that maintain one or more aspects of the nose, including dorsal, soft tissue, and tip preservation [9]. It is an emerging philosophy in rhinoplasty that has gained attention worldwide. As surgeons gain more experience with these methods, they seek innovative ways to incorporate preservation techniques into their primary rhinoplasty cases [10].

As the field of reconstructive and preservation rhinoplasty continues to evolve, future advancements will likely include minimally invasive techniques, bioengineered tissues, and the integration of artificial intelligence in surgical planning and execution. These developments promise to enhance the precision, safety, and outcomes of reconstructive rhinoplasty, offering patients improved solutions for both functional and aesthetic challenges.

The primary objective of this review is to comprehensively evaluate the advantages and disadvantages of various surgical techniques used in reconstructive and preservation rhinoplasty, focusing on both functional and aesthetic outcomes. By analyzing commonly employed methods, such as cartilage grafting, flap techniques, and the use of alloplastic materials, this review aims to identify the key factors contributing to successful outcomes, as well as potential complications. Additionally, the review will explore future directions in reconstructive and preservation rhinoplasty, including emerging technologies like 3D imaging, tissue engineering, and artificial intelligence, with the goal of understanding how these innovations may enhance current practices and improve surgical results and patient care.

## Review

Materials and methods

Study Selection

**Figure 1:**
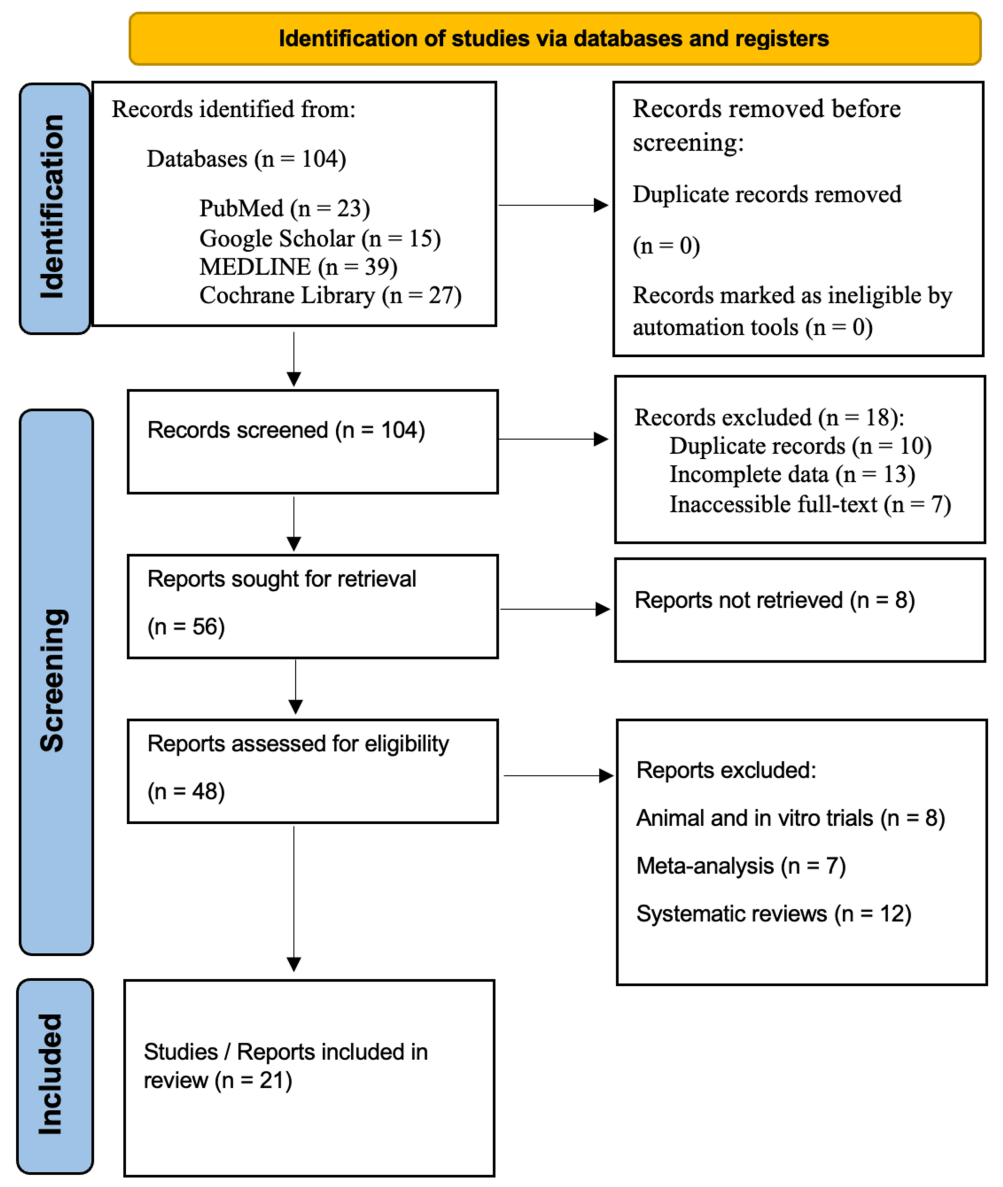
PRISMA flowchart: literature search and study selection PRISMA: Preferred Reporting Items for Systematic Reviews and Meta-Analyses [11]

In line with the Preferred Reporting Items for Systematic Reviews and Meta-Analyses (PRISMA) guidelines, a systematic review was conducted. Comprehensive searches were performed in the databases of PubMed, Google Scholar, MEDLINE, and the Cochrane Library for studies published over the last two decades, specifically from 2004 to 2024. The search utilized targeted keywords, including "Reconstructive rhinoplasty," "Preservation rhinoplasty," "Surgical techniques in reconstructive/preservation rhinoplasty," "Advantages of reconstructive/preservation rhinoplasty," "Disadvantages of reconstructive/preservation rhinoplasty," and "Reconstructive/preservation rhinoplasty and future directions." The review adhered to the PRISMA guidelines to ensure transparency and reproducibility throughout the research process, as shown in Figure 1.

Inclusion Criteria

The studies eligible for inclusion in this review had to meet specific criteria. First, they needed to involve human participants undergoing reconstructive and/or preservation rhinoplasty. Second, the studies had to report on scar quality. Additionally, they were required to present outcomes related to various factors, including the level of postoperative pain experienced by patients and their overall satisfaction. Lastly, the studies must have been published in English.

Exclusion Criteria

Certain studies were excluded from our selection criteria. Specifically, we omitted studies that did not provide sufficient data related to reconstructive or preservation rhinoplasty cases. Additionally, we did not include meta-analyses, reviews, or editorials that lacked original findings. Research conducted solely on animal models was also excluded. This rigorous selection process aimed to enhance the relevance and reliability of our review by concentrating exclusively on primary studies directly pertaining to the human population of interest.

Data Extraction

After selecting the studies based on the inclusion criteria, we proceeded with data extraction. The extracted data included information on the study design, sample size, patient demographics, scar quality (assessed using validated scar assessment scales), postoperative pain levels (measured with standardized pain scales), and postoperative satisfaction rates (evaluated through patient surveys and satisfaction scales). This comprehensive data extraction enabled a thorough and detailed analysis of the studies.

Results

Reconstructive Rhinoplasty

According to the data in Table 1, over the past decade, our research on clinical studies related to reconstructive rhinoplasty identified 11 studies that met our inclusion criteria. Among these, nine were observational studies, and two were case reports. These studies revealed the development of various techniques in this specialty. Key findings include: allogeneic cartilage is a viable alternative to autologous cartilage for both functional and reconstructive rhinoplasty; surgeons must understand the differences between primary and revision rhinoplasty; establishing a stable nasal framework using easily accessible, custom-fitted fresh frozen cadaveric rib grafts significantly enhances the outcomes of revision procedures; the free diced cartilage graft technique proves effective and easily reproducible for camouflage and augmentation in both aesthetic and reconstructive contexts; the comma strut provides reliable support for the nasal tip, with its dual-curved design being critical for defining the lobular-columellar angle and adjusting the supratip break; total nasal skeletal reconstruction, when performed by skilled surgeons on severely damaged noses, can lead to lasting functional and aesthetic improvements; the rainbow graft technique is safe and effective for revision rhinoplasty, particularly for restoring the nasal tip in complex secondary deformities; and bilateral intraoral mucosal flaps offer an effective solution for repairing vestibular stenosis following rhinoplasty and airway reconstruction, minimizing the need for revision procedures.

**Table 1:** Reconstructive rhinoplasty: a decade of clinical studies and advancements Data from [12-22].

References	Study design	Surgical technique	Objective of the study	Patients: size and patterns	Follow-up period	Evaluated parameters	Main outcomes	Conclusion
Read-Fuller et al. [12]	Observational study	Allogeneic cartilage for grafting	Assessing allogeneic cartilage grafting in patients undergoing reconstructive rhinoplasty.	24 patients who received allogeneic cartilage during functional or reconstructive rhinoplasty.	6 months	The patency of the internal and external nasal valves (INV and ENV, respectively) was assessed in patients who received Cartiform grafts, along with their cosmetic outcomes.	all patients exhibited open INVs and ENVs and reported significant improvement in nasal breathing; Patients rated their preoperative nasal breathing as a 4 out of 10 and their cosmetic appearance as a 3 out of 10; after surgery, these ratings improved to 9 out of 10; There were no complications reported, except for one case of superficial infection.	Allogeneic cartilage serves as a viable alternative to autologous cartilage in both functional and reconstructive rhinoplasty.
Hacker et al. [13]	Observational study	Multiple techniques	To assess the differences between the groups concerning indications, intraoperative methods, and postoperative results.	245 patients, consisting of 153 primary cases and 92 revision surgeries, were included for facial plastic surgery.	-	Complex reconstructive techniques, extracorporeal septoplasties, and extranasal grafts.	Nearly two-thirds of those undergoing revision experienced a combination of both functional and aesthetic concerns;	The treating surgeon must recognize the differences between primary and revision rhinoplasty.
Rohrich et al. [14]	Observational study	Fresh frozen rib cartilage grafts		226 patients who received open rhinoplasty utilizing fresh frozen rib cartilage grafts.	12.18 months	Warping, displacement, resorption, and infection.	Most patients had previously undergone one rhinoplasty procedure; The overall infection rate was 2.7 %, with the majority of cases managed successfully using antibiotics alone.	The outcomes of revision rhinoplasty are notably improved by establishing a stable nasal framework utilizing readily available, custom-fitted fresh frozen cadaveric rib grafts.
Kreutzer et al. [15]	Observational study	Free diced cartilage	To assess a newly developed technique for carefully dicing and accurately positioning free cartilage grafts obtained from the septum, rib, or ear cartilage.	446 patients (325 (group I) with free diced cartilage grafts as the only onlay, 73(group II) patients, the dorsal onlay was either fascia alone or in combination with free diced cartilage grafts and 48 (group III) received a dorsal augmentation with the classic diced cartilage in fascia technique)	7 months	-	The authors observed revision rates for dorsal irregularities within a 7-month postoperative period, which were 5.2%, 8.2%, and 25% for groups I, II, and III, respectively.	The authors' findings strongly corroborate their clinical experience, indicating that the free diced cartilage graft technique is an effective and easily reproducible approach for camouflage and augmentation in both aesthetic and reconstructive rhinoplasty.
You et al. [16]	Observational study	Comma-shaped columellar strut for nasal tip plasty	To introduce a new graft design for the columellar strut that resembles the shape of a comma.	165 patients received augmentation rhinoplasty surgery	12 mouths	The tip, columellar area, and corresponding angles, creating a visually appealing result that stands the test of time.	Patients' assessments of the overall improvements in their noses indicated a high level of satisfaction; Eight cases exhibited asymmetrical nostrils with a slight deviation of the columella, leading to the performance of minor revision surgeries; Three cases displayed dorsal graft warping, which was successfully addressed and no other significant complications occurred.	The comma strut offers dependable support for the nasal tip, and its dual curved structure is crucial for defining the lobular-columellar angle while also altering the supratip break.
Sazgar et al. [17]	Observational study	Skeletal reconstruction	Assessing the effectiveness of total nasal skeletal reconstruction for patients with severe post-rhinoplasty deformity after multiple revisions.	253 revision rhinoplasties	-	Septum, tip, dorsum, and side walls. The Nasal obstruction symptom evaluation (NOSE) instrument assessed nasal obstruction, while the Rhinoplasty outcome evaluation (ROE) instrument evaluated cosmetic results.	The patients had undergone an average of 3.2 previous rhinoplasties; Their mean ROE score was 6.36; Their mean NOSE score was 80.33; All patients underwent reconstruction of the septum, tip, dorsum, and side walls; One year postoperatively, the mean ROE score improved significantly to 17.27, and the mean NOSE score improved to 53.33.	Total nasal skeletal reconstruction, when performed by experienced surgeons on severely damaged noses, can achieve lasting functional and aesthetic improvements.
Park et al. [18]	Observational study	Osseocartilaginous cantilever graft	To describe and evaluate the results of modified osseocartilaginous rib cantilever grafting for rhinoplasty in patients with acute nasal bone fractures, focusing on both aesthetic and functional outcomes.	43 patients with acute nasal bone fractures, desiring both functional restoration and improved aesthetics, underwent osseocartilaginous rib grafting.	1 to 2 years	Dorsal skin flap, supraperichondrial and subperiosteal dissections.	Of the 43 patients, 37 (86%) reported "excellent" or "good" cosmetic outcomes. Three patients required secondary revision procedures. No donor-site morbidity was reported in any patient.	The cantilever technique, employing an osseocartilaginous rib graft, proves effective in achieving both anatomic reconstruction of the nasal dorsum and aesthetic tip refinement in acute nasal trauma patients seeking to enhance their profile during primary treatment.
Hanege et al. [19]	Observational study	Multiple techniques	To determine the prevalence of accompanying nasal pathologies in rhinoplasty patients and to identify whether any additional interventions were performed during surgery.	496 patients underwent rhinoplasty.	-	Septal deviation, inferior concha hypertrophy, unilateral or bilateral concha bullosa, nasal polyps, mucosal thickening, and retention cysts.	The Otorhinolaryngology clinic diagnosed 126 patients with septal deviation, inferior concha hypertrophy, unilateral or bilateral concha bullosa, nasal polyps, mucosal thickening, or retention cysts; A statistically significant difference was found in the prevalence of retention cysts between the clinics; The Otorhinolaryngology clinic had a significantly higher rate of retention cysts compared to the other clinic.	Additional nasal pathologies were frequently present in patients undergoing rhinoplasty operations. Hence, for a successful operation, it is essential to have Otorhinolaryngology consultation and detect accompanying pathologies in rhinoplasty cases.
Bracaglia et al. [20]	Case report	Rainbow graft for tip reconstruction	To present the authors' experience with the rainbow graft.	21 patients who had undergone revision rhinoplasty	1 to 12 years	The presence of tip deformities, including asymmetry, dome angulation with cartilage disruption, alar pinch, alar retraction, and underprojection or overprojection.	Projection was successfully corrected in all patients; There were no instances of graft infection, malposition, or resorption; The corrections remained stable throughout the follow-up period; The mean postoperative ROE score was 80.75 ± 6.24, with the improvement being statistically significant.	The rainbow graft is a safe and effective technique for revision rhinoplasty, particularly indicated for the complete restoration of nasal tip appearance in cases of complex secondary deformities.
Dickinson et al. [21]	Case report	Bilateral intraoral mucosal flaps for repair of vestibular stenosis following rhinoplasty and airway reconstruction	To introduce a straightforward method and technique for repairing vestibular stenosis that is easily replicable.	A female patient underwent septorhinoplasty to​ diminish the size of her nose and enhance breathing.	-	-	The lower lateral cartilages were rotated upwards using a combination of a tip rotation suture and a tongue-in-groove technique, followed by an excision of the nostril sill; The patient was satisfied with the aesthetic outcome.	Bilateral intraoral mucosal flaps are an effective solution for repairing vestibular stenosis following rhinoplasty and airway reconstruction, thereby minimizing the necessity for revision rhinoplasty.
Şahin et al. [22]	Observational study	Different middle vault reconstruction techniques	To assess the outcomes of various middle vault rhinoplasty techniques using multiple patient-reported outcome measures (PROMs) and to compare their differences based on these findings.	129 patients.	12 months	-	All techniques demonstrated significant improvements across all PROMs, with the exception of the dorsal preservation rhinoplasty (DPR) with high strip (DPRwHS) in the NOSE measure; No significant differences were noted between the short-term and longer-term postoperative results within the DPR groups, in contrast to the structural techniques.	In this comparative study of various middle nasal vault rhinoplasty techniques, we found no difference in the enhancement of patient-reported outcomes from the DPR techniques, observed from as early as 2 months up to 1 year postoperatively.

Preservation Rhinoplasty

The outcomes of our review on preservation rhinoplasty, presented in Table 2, highlight clinical studies from the past decade. Our bibliographic research identified nine observational studies and one case report focusing on this topic. These studies reveal the development of various techniques and approaches related to preservation rhinoplasty. Notably, the findings indicate that the let-down and push-down techniques yield improved results, providing surgeons with diverse options. The unilateral lateral crural turn-up flap has emerged as an effective method for correcting cartilaginous nasal sidewall asymmetries in patients with crooked noses treated via dorsal preservation rhinoplasty (DPR). Additionally, the dorsal roof flap technique allows for the preservation of the nasal dorsum while effectively removing the dorsal hump, reducing the likelihood of hump recurrence. DPR has also proven effective for addressing Asian hump noses. Furthermore, reconstructing nasal ligaments, including the scroll, septocolumellar, and Pitanguy ligaments-can help maintain nasal tip projection and rotation over time. Techniques such as MSSM or Z-flap cartilage manipulation in DPR have shown significant improvements in nasal aesthetics, breathing, and sleep, as indicated by patient feedback on validated assessment tools. Therefore, rhinoplasty surgeons should consider incorporating dorsal preservation techniques into their surgical repertoire, rather than relying solely on the Joseph reduction method or open structure rhinoplasty. This represents a significant shift in rhinoplasty practices, as surgeons increasingly evaluate the feasibility of preserving anatomical structures in each case. The choice between open and closed approaches should be guided by the specific tip and dorsal deformities present, with closed rhinoplasty being preferable for patients with thin skin, minimal dorsal modifications, and the preservation of osseocartilaginous structures.

**Table 2:** Decade-long clinical studies on preservation rhinoplasty Data from [23-32].

References	Study design	Surgical technique	Objective of the study	Patients: size and patterns	Follow-up period	Evaluated parameters	Main outcomes	Conclusion
Kosins [23]	Observational study	Open and closed preservation rhinoplasty	The author initially utilized an open approach but decided that a closed approach could be advantageous for certain patients.	162 primary rhinoplasty cases were examined in a retrospective study.	12 months	Dissection planes, preservation of the dorsum (DP) compared to component reductions, surface techniques versus foundational DP techniques, and open approach versus closed approach.	83 patients had their dorsal soft tissue envelope preserved; Every patient who underwent a closed approach also had preservation of this envelope; Among the 67 patients who received dissection planes (DP), 38 utilized surface techniques, while 29 employed impaction techniques; 33 patients underwent structural rhinoplasty, which included piezoelectric osteotomies and mid-vault reconstruction, all performed using an open approach.	The choice between open and closed approaches depends on the specific tip and dorsal deformities present. A closed rhinoplasty (PR) is preferred for patients with thin skin, minimal dorsal modifications, preservation of osseocartilaginous structures. Conversely, an open rhinoplasty is recommended for cases requiring extensive dorsal modifications, those with S-shaped nasal bones, complex tip deformities, and situations that involve tip augmentation.
Kosins [24]	Observational study	Dorsal preservation rhinoplasty with cartilage conversion techniques	presents a collection of 100 primary rhinoplasties focused on dorsal preservation, with a particular emphasis on cartilage-only preservation techniques.	226 primary rhinoplasty cases were studied retrospectively	12 months	Reduction volume, alignment procedures, nasal bone surgery encompassing various types of piezoelectric osteotomies and piezoelectric rhinosculpture, along with stabilization methods.	57 patients underwent the sub-dorsal strip technique, with 39 receiving the cartilage-only push-down technique and 57 undergoing cartilage modification. The average lowering achieved was 4.5 mm for the sub-dorsal strip technique, 2.5 mm for the cartilage-only push-down technique, and 2 mm for the cartilage modification technique. None of the patients required revision surgery for their dorsum.	Preservation rhinoplasty represents a significant shift in the approach to rhinoplasty. Over time, surgeons will increasingly consider whether it is possible to preserve anatomical structures in every case. Dorsal preservation has proven to be a dependable technique when patients are appropriately selected.
Taş [25]	Observational study	Dorsal roof technique for dorsum preservation	Presenting the dorsal roof technique for both reducing and narrowing the nasal dorsum during rhinoplasty.	52 patients underwent septorhinoplasty surgery using the dorsal roof technique.	12 months	Pyramidal angles	No irregularities or residual humps were detected; The procedure resulted in significant narrowing; Patients reported high satisfaction, and no functional or aesthetic complications were observed.	The dorsal roof technique provides a non-resection approach to addressing the wide dorsum, wide nasal base, and dorsal hump, preserving the integrity of the dorsal bone-cartilage complex, making it a preferred option over resection or camouflage.
Saban et al. [26]	Observational study	Dorsal preservation	Presenting the senior author's current operative technique for dorsal preservation in reduction rhinoplasty, drawing on experience from 320 clinical cases.	320 patients had a dorsal preservation operation (DPO)	2 years and 5 months	-	No dorsal irregularities or inverted-V deformities were observed. Among our 44 personal revision cases, 27 patients (8.74%) had previously undergone DPO. Of these, 16 patients required tip revisions without further dorsal surgery. The remaining 11 patients presented with either hump recurrence, lateral deviation of the dorsum, or widening of the middle third, requiring simple surgical revision.	Rhinoplasty surgeons should consider adding dorsal preservation techniques to their surgical repertoire, rather than relying solely on the Joseph reduction method or open structure rhinoplasty.
Sozansky Lujan et al. [27]	Observational study	Let-down dorsal preservation rhinoplasty	To assess the differences in patient-reported perceptions of nasal aesthetics, nasal breathing, and sleep quality, and to compare the outcomes of two distinct septal cartilage manipulation techniques in patients undergoing preservation rhinoplasty.	52 patients underwent dorsal preservation rhinoplasty	12 months	The modified subdorsal strip method (MSSM) or subdorsal Z-flap are evaluated both before and after surgery using established assessment tools, including the nose obstruction symptom evaluation (NOSE), sinonasal outcome test (SNOT-22), Standardized Cosmesis and Health Nasal Outcomes Survey (SCHNOS), and the Epworth Sleepiness Scale (ESS).	The majority of these patients reported significant improvements at 1, 3, 6, and 12 months postoperatively, as indicated by paired t-tests applied to NOSE, SNOT-22, SCHNOS, and ESS scores; No significant difference was found between the MSSM and Z-flap techniques.	Dorsal preservation rhinoplasty using either the MSSM or Z-flap cartilage manipulation technique can result in significant enhancements in nasal aesthetics, nasal breathing, and sleep, as indicated by patient responses on validated assessment tools.
Ali et al. [28]	Observational study	Ligament preservation in open rhinoplasty	To evaluate the impact of preserving the nasal ligaments (specifically the scroll, septocolumellar, and Pitanguy ligaments) in open rhinoplasty procedures.	32 patients underwent open rhinoplasty with ligament preservation following specialized training conducted on five cadaver specimens.	-	-	All patients experienced enhanced aesthetic and functional outcomes in the early postoperative period, with sustained preservation of tip projection and results over time; None of the patients required secondary revision surgery due to issues such as tip dropping or malrotation; both objective findings and subjective evaluations were satisfactory for patients and surgeons alike.	The possibilities for refining nasal surgery are endless. This study indicates that reconstructing nasal ligaments, including the scroll, septocolumellar, and Pitanguy ligaments, may effectively preserve nasal tip projection and rotation over an extended period.
Jin et al. [29]	Case report	Dorsal preservation rhinoplasty	To discuss the feasibility, surgical results, and technical aspects of dorsal preservation rhinoplasty for correcting hump noses in Asian individuals.	9 patients who underwent primary dorsal preservation rhinoplasty to correct hump noses.	8.3 months	Nasofrontal angle (NFrtA), nasofacial angle (NFcA), nasolabial angle (NLA), rhinion angle (RA), radix height, dorsal hump height, nasal length, and tip projection length.	In three cases, bony step-off camouflage was necessary at the transverse osteotomy site; post-surgery, significant changes were observed in both the nasofacial and rhinion angles; hump reduction was successfully achieved in all cases, with no occurrences of recurrence or saddle nose, and no major complications arose; Each patient expressed satisfaction with the aesthetic and functional outcomes.	Dorsal preservation rhinoplasty appears to be an effective approach for addressing Asian hump noses.
Tuncel et al. [30]	Observational study	Dorsal preservation surgery: a novel modification for dorsal shaping and hump reduction	To introduce a novel dorsal roof flap (DRF) technique aimed at modifying the nasal hump and dorsum while minimizing morbidity.	25 patients received one of two types of nasal DRFs, depending on the composition of their humps.	10.3 months	Preoperative and postoperative nasolabial (NLA) and nasoglabellar angles (NGA)	Out of the observed humps, 22 were categorized as V-shaped and 3 as S-shaped; the composition of the humps included cartilage in 5 instances, bone in 7 instances, and a combination of both in 13 instances; a DRF-c was utilized for the 5 cases featuring a cartilaginous hump, while a DRF-oc was employed for the remaining 20 cases.	The dorsal roof flap technique enables the preservation of the nasal dorsum while effectively removing the dorsal hump and reducing the likelihood of hump recurrence.
Erdal et al. [31]	Observational study	Lateral crural turn-up flap	To present a flap designed for use in cases of crooked noses treated with dorsal preservation rhinoplasty, as well as to demonstrate potential modifications of this flap.	8 patients who had lateral crural turn-up flap due to crooked nose.	12 months	Standardized front-view photographs taken before and after surgery, and rated by two plastic surgeons for their effectiveness in correcting midvault nasal sidewall asymmetries.	The surgeons assigned an average score of 4.18 for the correction of midvault nasal sidewall asymmetries; The mean rhinoplasty outcome evaluation (ROE) score was 89, indicating that all patients were satisfied based on their ROE assessments; There were no significant complications reported.	The unilateral lateral crural turn-up flap appears to be an effective technique for correcting cartilaginous nasal sidewall asymmetries in cases of crooked nose treated with dorsal preservation rhinoplasty.
Öztürk [32]	Observational Study	Semi-let-down and semi-push-down preservation techniques	Several approaches have been established in which the let-down and push-down techniques were utilized according to the patients' needs.	64 patients who were eligible for the new approaches underwent either push-down or let-down techniques for hump reduction.	19.2 months	-	Before surgery, the median ROE score was 61.6; this improvement in the ROE score was statistically significant; the satisfaction rate was notably high at 93.75% according to the ROE scale.	These methods for the let-down and push-down techniques will yield improved outcomes and provide surgeons with varied options.

Discussion

Reconstructive Rhinoplasty

Key findings from our review highlight the viability of allogeneic cartilage as an alternative to autologous cartilage for both functional and reconstructive procedures. Furthermore, surgeons must understand the distinct considerations involved in primary versus revision rhinoplasty. Innovative graft techniques, such as the use of custom-fitted fresh frozen cadaveric rib grafts for creating a stable framework, the free diced cartilage graft technique for effective camouflage and augmentation, and the comma strut for reliable tip support, have demonstrated improved outcomes. These studies collectively underscore ongoing advancements in reconstructive rhinoplasty, offering surgeons and patients a broader spectrum of options for achieving optimal functional and aesthetic results.

In 2017, Bogari et al. introduced an innovative rhinoplasty technique that incorporates osteochondral rib as an autogenous implant, drawing inspiration from the ancient Chinese architectural method known as DouGong. The authors present findings from a study of 288 patients treated at their institution, where patient data and preoperative and postoperative three-dimensional computed tomography scans were analyzed using Mimics software (Materialise, Leuven, Belgium). A distinctive feature of these implants is that the connection between the nasal dorsum and the columella strut is secured without the use of screws, stitches, or K-wires. This approach has proven advantageous, as it reduces the need for fixation techniques while also improving the nasofrontal angle, nasolabial angle, and columella length. After conducting follow-up assessments of their patients, the authors advocate for the adoption of this DouGong-inspired technique to enhance the overall quality and outcomes of corrective rhinoplasty [33].

Advantages of Reconstructive Rhinoplasty

Reconstructive rhinoplasty provides numerous benefits, particularly in restoring both the functional and aesthetic aspects of the nose [34]. One of the main advantages is the improvement of nasal breathing, especially in cases where structural damage or congenital deformities have obstructed airflow. Additionally, this procedure enhances facial symmetry and overall appearance, significantly boosting a patient's self-esteem and psychological well-being [35]. With advanced surgical techniques, including cartilage grafts and flap methods, surgeons can achieve precise reshaping of the nose, resulting in more natural and long-lasting outcomes. Reconstructive rhinoplasty also offers the opportunity to correct previous surgical complications or trauma-related deformities, giving patients renewed confidence in their appearance. Furthermore, the integration of modern technologies, such as 3D imaging and printing, allows for increasingly customized surgeries tailored to each patient's unique anatomy, enhancing both the accuracy of the procedure and the overall results [36].

Disadvantages of Reconstructive Rhinoplasty

While reconstructive rhinoplasty offers numerous advantages, it also carries some potential drawbacks that patients should consider. One significant disadvantage is the complexity of the procedure, which requires a high level of surgical expertise. This complexity can lead to longer and more technically demanding operations, increasing the risk of complications such as infection, scarring, graft displacement, or unsatisfactory aesthetic outcomes [37]. Patients may also experience prolonged swelling and an extended recovery period, during which they might face discomfort and temporary breathing difficulties. Revision cases present an additional challenge, as previous surgeries have altered the nasal anatomy, making it harder for surgeons to achieve optimal results. The use of grafts, whether from the patient's own tissue or synthetic materials, also carries the risk of resorption or rejection, potentially necessitating further corrective surgeries [38]. Finally, the cost of reconstructive rhinoplasty can be substantial, and some patients may not achieve their desired outcome, leading to the need for additional procedures or revisions.

Preservation Rhinoplasty

Our review identified ten studies on preservation rhinoplasty, revealing various techniques, with the let-down and push-down methods showing improved outcomes. The unilateral lateral crural turn-up flap and dorsal roof flap technique emerged as effective approaches for correcting nasal asymmetries and preserving the nasal dorsum. Surgeons are increasingly incorporating dorsal preservation techniques, marking a shift away from traditional methods, particularly in cases with minimal dorsal modifications.

Preservation rhinoplasty maintains and expands the internal nasal valve angle, resulting in high levels of patient satisfaction, and presents a promising option for both functional and aesthetic improvements in rhinoplasty [39]. The basis of this technique is built upon recent anatomical studies, innovative tip suture techniques, and enhanced surgical methods [40]. Interestingly, DPR is practiced worldwide, with Turkey and Mexico emerging as prominent regions. However, its global application varies by area due to inconsistent outcomes, predictability, and complications [41]. This technique has also been effectively applied to Andean mestizo patients, whose distinctive anatomical characteristics had not been previously examined with this technique. A recent study demonstrates that modifications in the surgical approach, such as the use of a reinforced columellar strut, can yield similarly positive outcomes [42]. Remarkably, the cornerstone of traditional rhinoplasty is dorsal resection, which disrupts the keystone area and necessitates immediate osteotomies and midvault reconstruction. Today, dorsal reconstruction in secondary cases is the primary reason for the majority of rib graft reconstructions [43].

Advantages of Preservation Rhinoplasty

Preservation rhinoplasty offers several notable advantages over traditional methods by focusing on maintaining and optimizing existing nasal structures rather than extensive resection. This approach often results in a more natural appearance and improved preservation of nasal function, as it minimizes disruption to critical supportive tissues [44]. By conserving the underlying cartilage and bone, preservation rhinoplasty can lead to quicker recovery times and a reduced risk of complications such as graft resorption or deformities [45]. One of the primary advantages is its ability to maintain the nose's natural anatomy while achieving both aesthetic and functional improvements. Preservation rhinoplasty typically involves less trauma to the nasal framework, which can enhance postoperative stability and the longevity of results [46]. Additionally, the minimally invasive nature of this technique allows for more precise adjustments and a more predictable outcome, often leading to higher patient satisfaction. Furthermore, by minimizing trauma to nasal structures, preservation rhinoplasty can result in quicker recovery times for patients [47].

In a recent study by Neves and Arancibia-Tagle (2021), the authors concluded that DPR is a safe and natural procedure for deprojecting the nasal pyramid in appropriately selected patients. Careful patient selection is crucial to minimizing drawbacks and complications. However, even with careful planning, some issues and limitations may arise, requiring anticipation, examination, and management. To achieve a predictable, accurate, and aesthetically pleasing outcome, it is best to approach the nasal pyramid segment by segment, analyzing specific characteristics and applying corresponding solutions [48].

Disadvantages of Preservation Rhinoplasty

While preservation rhinoplasty offers benefits such as maintaining nasal structure and reducing recovery time, it also has some disadvantages [49]. A significant drawback is the limited scope for major structural changes, as the technique primarily focuses on conserving existing anatomy [50]. This can be problematic for patients requiring extensive reshaping or correction of severe nasal deformities. Additionally, the procedure demands high surgical precision and expertise, which may not be widely available, increasing the risk of suboptimal outcomes. Lastly, because preservation rhinoplasty is a relatively newer approach, long-term data on outcomes and potential complications are still limited, making it a less predictable option compared to traditional methods [51].

Future Directions

Over the past decade, significant progress has been made in our understanding of nasal anatomy, particularly in its relationship to nasal aesthetics and surgical techniques. Two key areas of interest are the composition of the soft tissue envelope, including the nasal ligaments, and the osseocartilaginous vault. Although often overlooked in the past, nasal ligaments play a crucial role in both the functional and aesthetic aspects of the nose [52]. Future innovations show great promise for advancing both reconstructive and preservation rhinoplasty techniques.

A recent study described a novel tip plasty procedure involving the sliding of the cephalic portion of the alar cartilage underneath its caudal portion to preserve the scroll area, as illustrated using 3D modeling software. This study presents the prospective case series, including NOSE and PNIF scores to objectively assess functional outcomes. The authors concluded that the sliding alar cartilage (SAC) is an effective and straightforward method for defining the tip while preserving nasal airway function by safeguarding the essential anatomical scroll area [53]. 3D printing enables surgeons to customize grafts and guides for precise reconstruction or refinement of individual nasal structures, improving predictability of surgical outcomes [54]. Additionally, the integration of artificial intelligence (AI) in preoperative assessments and intraoperative guidance is expected to improve precision and minimize complications. Tissue engineering and regenerative medicine also hold considerable promise, with ongoing research focused on developing bioengineered grafts that could replace traditional cartilage or synthetic materials, resulting in more natural and durable outcomes [41].

Structural preservation rhinoplasty (a hybrid approach) involves blending dorsal preservation techniques with structural grafting to enhance results for the nasal dorsum, while utilizing structural grafting techniques to address the lower third of the nose [55]. In structural preservation rhinoplasty, dorsal preservation techniques are used for the upper two-thirds of the nose in suitable primary rhinoplasty cases, while structural cartilage grafting is employed to enhance dorsal aesthetic lines and shape the nasal tip. Dorsal preservation techniques are particularly indicated for primary rhinoplasty cases with specific anatomical criteria, including a V-shaped dorsal hump, standard radix height, and uncomplicated axis deviations [56]. Based on the ROE scale, Öztürk (2022) reported excellent patient satisfaction in 91.6% of cases involving hybrid preservation rhinoplasty, combining mix-down and semi-let-push-down techniques. This innovative approach is expected to be suitable for select patients and easy for surgeons to perform. Additionally, patients with combined hump and deviation deformities are likely to benefit from the hybrid dorsal preservation technique [57].

Minimally invasive procedures like endoscopic rhinoplasty aim to reduce trauma to nasal tissues and shorten recovery times. The future of dorsal augmentation rhinoplasty promises to deliver more personalized, precise, and patient-focused methods, which will ultimately transform the standards in cosmetic surgery [58]. Additionally, the application of artificial intelligence in preoperative planning and postoperative analysis facilitates personalized care decisions and evaluations of postoperative changes over time. Case studies highlighted in this review demonstrate how these innovations have already benefited patients through techniques like endoscopic rhinoplasty, providing enhanced precision and satisfaction while preserving nasal function. Moving forward, continued integration of these technologies into rhinoplasty practices could propel the field to new heights, optimizing both cosmetic results and patient well-being. The results of the current review indicate that laser-assisted rhinoplasty is a promising, feasible, and safe option with no major complications. Its aesthetic and functional outcomes are comparable to traditional rhinoplasty, but it offers greater intraoperative precision, enhanced patient satisfaction, a cleaner surgical field, and reduced bleeding.

Wo et al. (2020) emphasize the necessity of establishing clear guidelines and nomenclature for defining rhinoplasty, septoplasty, and septorhinoplasty to accurately assess outcomes. Given the variety of available techniques, surgeons must rely on data to enhance their practices and effectively meet patient needs. Consequently, it is essential to outline the objectives of each technique clearly and create a standardized framework that separates functional outcomes from cosmetic results [59]. Furthermore, rhinoplasty simulators have the potential to enhance trainees' confidence and knowledge in the operating room, though they require further refinement, validation, and assessment to ensure broader implementation and acceptance [60].

Clinical Impact

The analysis of reconstructive and preservation rhinoplasty techniques offers important insights that can inform clinical decision-making. By recognizing the distinctions in patient demographics, surgical objectives, and technical methods for each procedure, practitioners can better assess individual cases to choose the most suitable rhinoplasty approach. The reconstructive preservation framework can facilitate preoperative planning and help manage patient expectations. Additionally, the future innovations discussed provide practical strategies for incorporating minimally invasive techniques, tailored tools, and AI-driven analytics into everyday practice, further improving surgical outcomes and enhancing patient-centered care. Overall, this review provides rhinoplasty specialists with valuable frameworks and perspectives relevant to their clinical practice.

## Conclusions

In summary, this paper has examined the differences between reconstructive and preservation rhinoplasty techniques. Reconstructive rhinoplasty is recommended for patients with significant nasal deformities or structural damage resulting from trauma or medical conditions. This technique often involves the use of cartilage grafts and may require an external approach to effectively reconstruct the nasal framework. In contrast, preservation rhinoplasty is ideal for patients primarily seeking cosmetic enhancements, focusing on refining the shape and tip of the nose. This method adopts a more conservative internal approach, reshaping the nose without fundamentally altering its existing structure. While both procedures can improve facial aesthetics and enhance breathing, reconstructive rhinoplasty is focused on rebuilding the nose, whereas preservation rhinoplasty emphasizes subtle enhancements and refinements. Therefore, selecting the appropriate rhinoplasty technique requires a careful assessment of each patient's specific clinical needs and desired outcomes.
